# Supplementary material for: Rapid detection of ERG11 polymorphism associated azole resistance in Candida tropicalis
Source: PLoS One. 2021 Jan 13;16(1):e0245160. doi: 10.1371/journal.pone.0245160 (PMC7806177; doi:10.1371/journal.pone.0245160)
Supplement: S1 File — (DOCX) [file pone.0245160.s001.docx]

**Rapid detection of *ERG11* polymorphism associated azole resistance in *Candida tropicalis***

Saikat Paul, Rajneesh Dadwal, Shreya Singh, Dipika Shaw, Arunaloke Chakrabarti, Shivaprakash M. Rudramurthy, Anup K Ghosh*

Department of Medical Microbiology, Postgraduate Institute of Medical Education and Research (PGIMER), Chandigarh - 160012, India.

*** Corresponding author**.

**Correspondence**: **Dr. Anup K Ghosh**
 Additional Professor

Department of Medical Microbiology,

Postgraduate Institute of Medical Education and Research (PGIMER),

Chandigarh 160012, India.
 Email: anupkg3@gmail.com

Tel.: +91 172 2755156.

Fax: +91 172 2744401.

**S1 Table. Sequences of overlapping primers for mutation analysis of *ERG11* gene**

| **Primer name** | **Sequence (5'->3')** | **Starts** | **Stops** | **Product length** |
| --- | --- | --- | --- | --- |
| *ERG11*-F1 | TCACAGTTATAGACCCACAAGG | -71 | -50 | 878 |
| *ERG11*-R1 | TCACCGCTTTCTCTTCTTCTCT | 806 | 785 |  |
| *ERG11*-F2 | AAGGTTTCACCCCAATCAACTT | 677 | 698 | 1113 |
| *ERG11*-R2 | CGACTGAAACGTATACCGCGA | 1789 | 1769 |  |

F1 and F2 = Forward primer for fragment 1 and 2; R1 and R2 = Reverse primer for fragment 1 and 2

**S2 Table. Details of the azole susceptible *C. tropicalis* isolates used**

| **NCCPF ID** | **GenBank accession number** | **Source of isolates** | **Fluconazole MIC (mg/L)** | **Voriconazole MIC (mg/L)** | **Itraconazole MIC (mg/L)** | **Posaconazole MICs (mg/L)** |
| --- | --- | --- | --- | --- | --- | --- |
| 420214 | MW015968 | Blood | 1 | 0.03 | 0.06 | 0.06 |
| 420215 | MW015969 | Blood | 0.5 | 0.06 | 0.12 | 0.03 |
| 420203 | MW015970 | Blood | 1 | 0.12 | 0.12 | 0.06 |
| 420200 | MW015971 | Blood | 0.5 | 0.03 | 0.03 | 0.06 |
| 420212 | MW015972 | Blood | 0.5 | 0.25 | 0.12 | 0.25 |
| 420210 | MW015973 | Blood | 0.5 | 0.03 | 0.06 | 0.06 |
| 420199 | MW015974 | Blood | 1 | 0.03 | 0.12 | 0.12 |
| 420205 | MW015975 | Ascitic Fluid | 1 | 0.25 | 0.12 | 0.06 |
| 420204 | MW015976 | Blood | 0.5 | 0.06 | 0.12 | 0.03 |
| 420198 | MW015977 | Blood | 0.5 | 0.12 | 0.06 | 0.03 |
| 420196 | MW015978 | Blood | 1 | 0.12 | 0.06 | 0.12 |
| 420197 | MW015979 | Blood | 0.5 | 0.06 | 0.06 | 0.03 |
| 420202 | MW015980 | Blood | 1 | 0.06 | 0.06 | 0.03 |
| 420206 | MW015981 | Blood | 0.5 | 0.06 | 0.03 | 0.03 |
| 420207 | MW015982 | Blood | 1 | 0.25 | 0.25 | 0.12 |
| 420208 | MW015983 | Blood | 1 | 0.25 | 0.12 | 0.12 |
| 420209 | MW015984 | Blood | 1 | 0.12 | 0.12 | 0.25 |
| 420211 | MW015985 | Blood | 0.5 | 0.03 | 0.03 | 0.03 |
| 420213 | MW015986 | Blood | 1 | 0.06 | 0.12 | 0.25 |

**
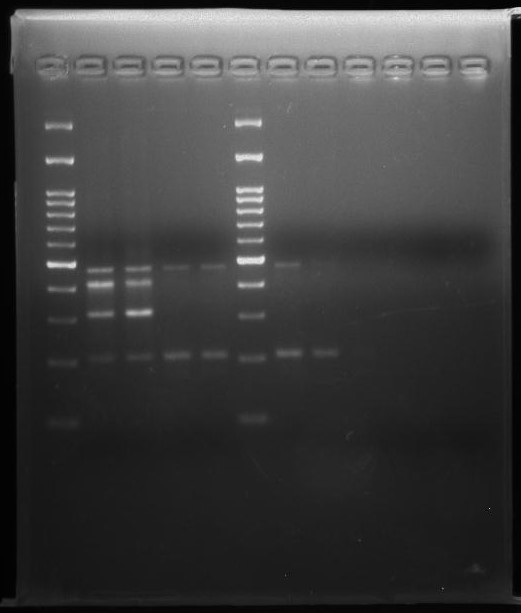
**

**S1 Fig. Original uncropped and unadjusted gel images of Fig 1 for the analysis of A395T mutation among resistant (R) and susceptible (S) isolates.**

**
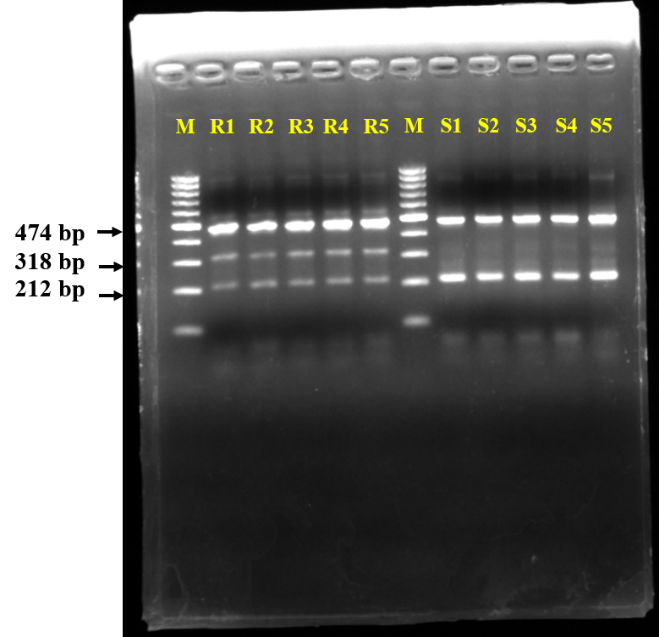
**

**S2 Fig. T-ARMS-PCR analysis of A395T mutation among resistant (R) and susceptible (S) isolates.** Figure representing the agarose gel electrophoresis of 5 resistant and 5 susceptible isolates. M: 100 bp molecular weight markers

**
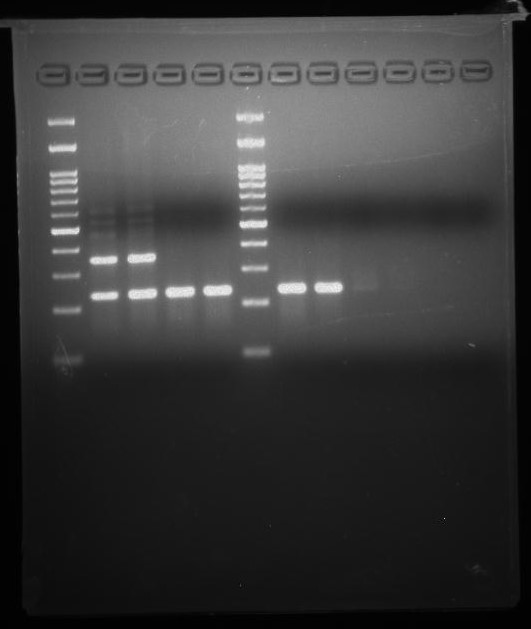
**

**S3 Fig. Original uncropped and unadjusted gel images of Fig 2 for the analysis of C461T mutation among resistant (R) and susceptible (S) isolates.**

**
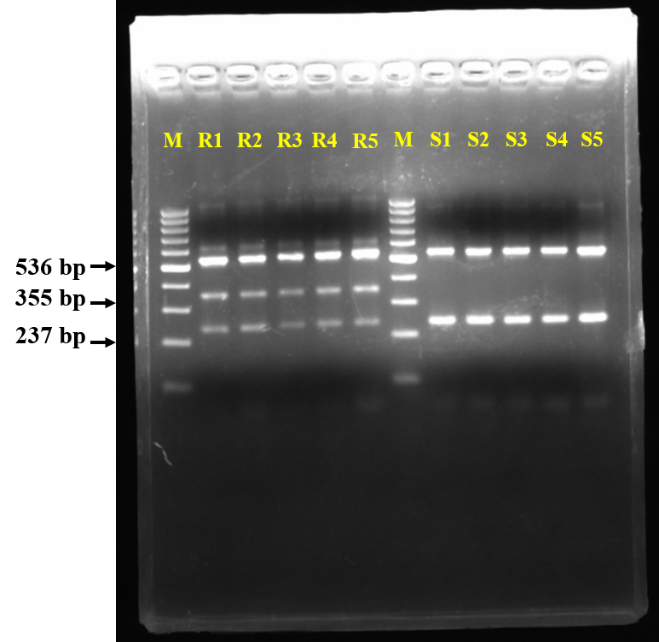
**

**S4 Fig. T-ARMS-PCR analysis of C461T mutation among resistant (R) and susceptible (S) isolates.** Figure representing the agarose gel electrophoresis of 5 resistant and 5 susceptible isolates. M: 100 bp molecular weight markers

**
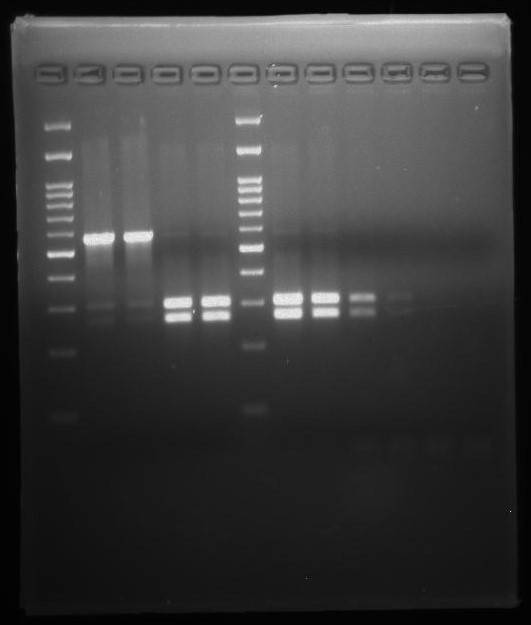
**

**S5 Fig. Original uncropped and unadjusted gel images of Fig 3 for the analysis of C461T mutation screening among resistant (R) and susceptible (S) isolates.**


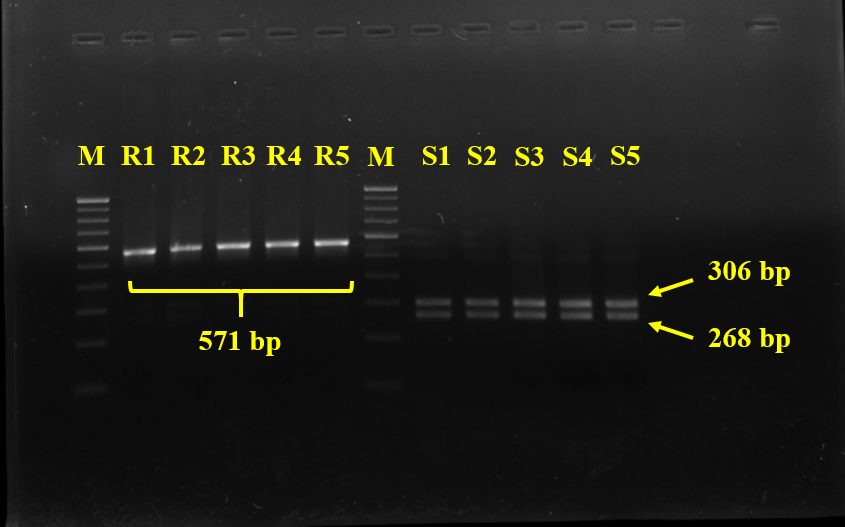


**S6 Fig. RSM analysis for *ERG11* mutation screening.** Figure representing the agarose gel electrophoresis of 5 resistant and 5 susceptible isolates. M: 100 bp molecular weight markers

**
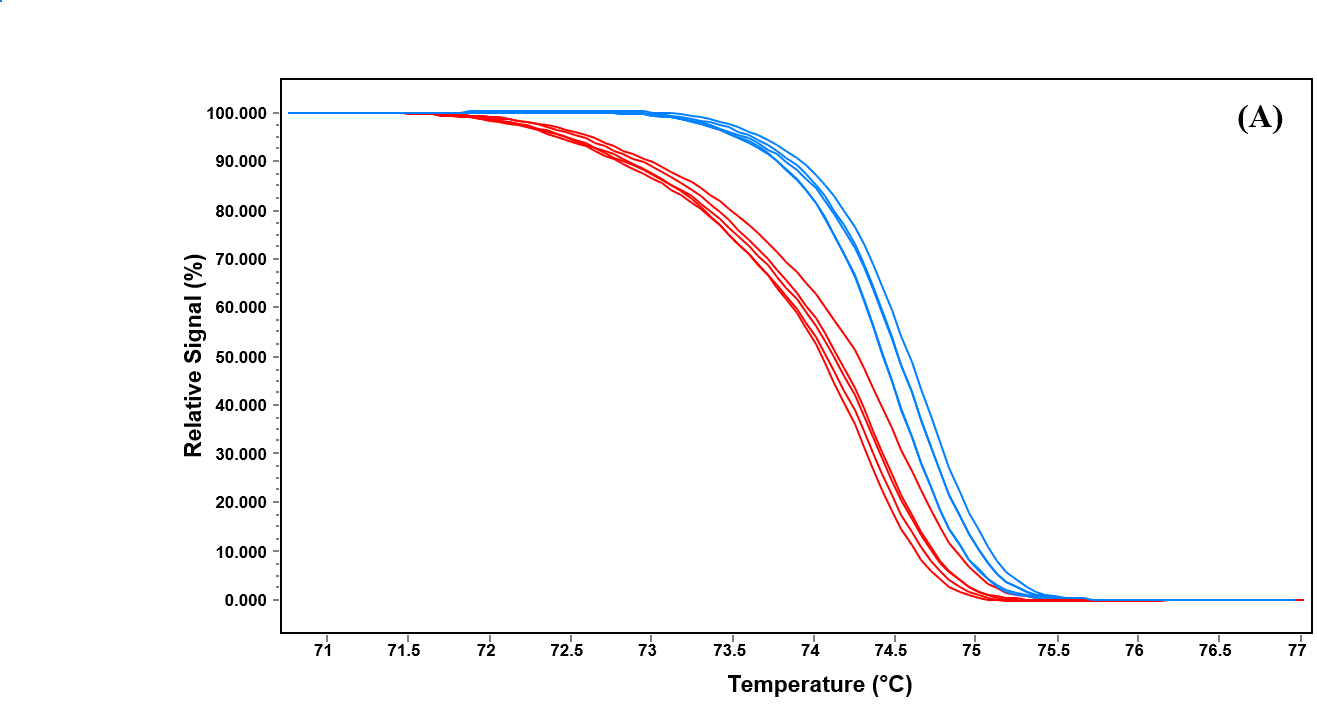
**

**S7 Fig. HRM analysis of the *ERG11* gene of *C. tropicalis*.** Graph representing normalized melting curve of 5 resistant and 5 susceptible isolates. Red curves resistant variant and blue curves susceptible variant.

**
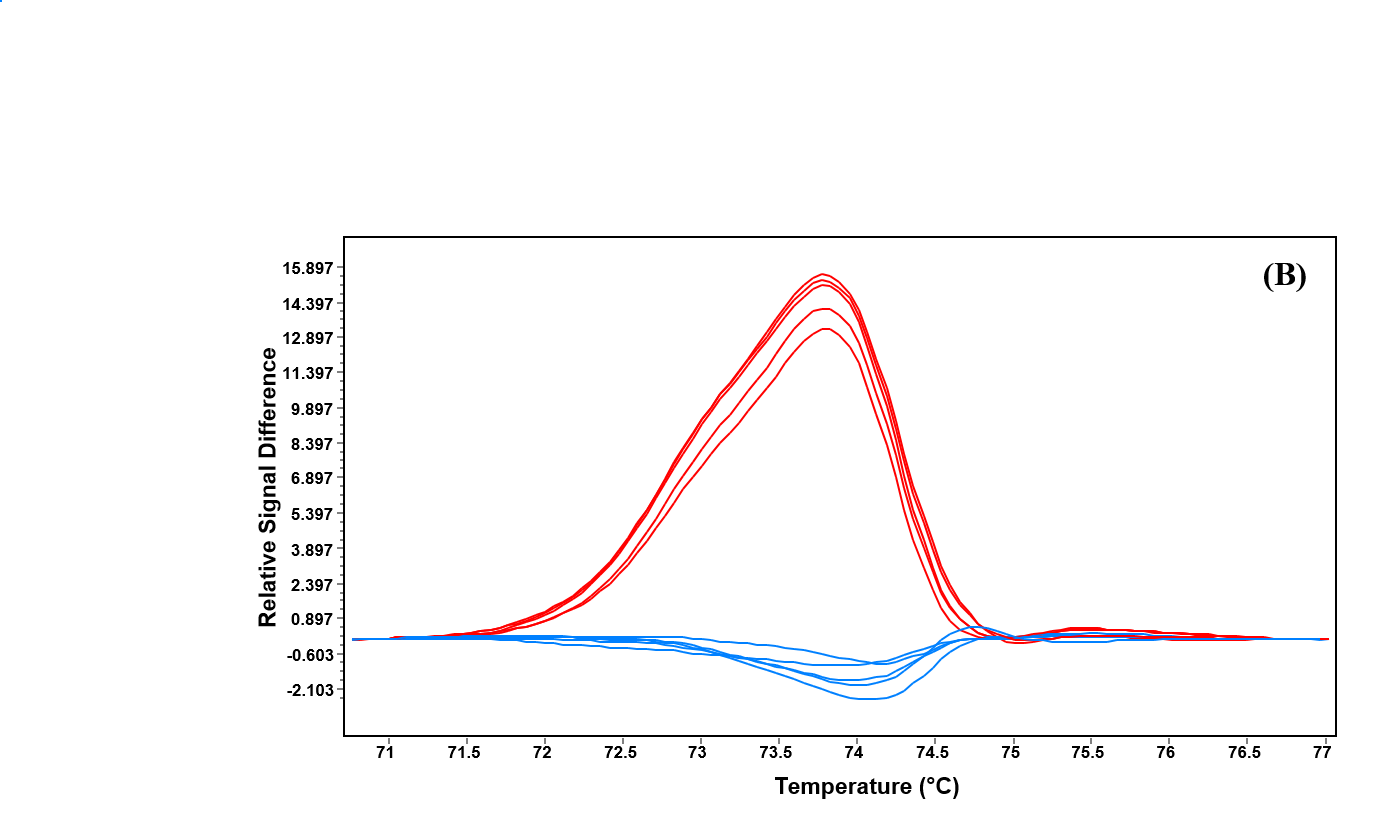
**

**S8 Fig. HRM analysis of the *ERG11* gene of *C. tropicalis*.** Difference plot presenting two variants of the *ERG11* gene fragment among the 5 resistant and 5 susceptible isolates. Red curves resistant variant and blue curves susceptible variant.
