# Supplementary material for: Rapid detection of ERG11 polymorphism associated azole resistance in Candida tropicalis
Source: PLoS One. 2021 Jan 13;16(1):e0245160. doi: 10.1371/journal.pone.0245160 (PMC7806177; doi:10.1371/journal.pone.0245160)
Supplement: Attachment — Submitted filename: Response to Reviewers.docx [file pone.0245160.s003.docx]

**Editor comments:**

**Comment:** Please consider and offer a response to Reviewer #1. If possible, address this issue in a short comment in discussion of limitations of your study.

**Response:** Thank you for considering our manuscript in your esteemed journal. We have tried our best to address all the issues raised by the Reviewer #1. We have addressed this issue in a short comment in discussion as the limitations of our study in the revised manuscript.

**Comment:** Please include the following items when submitting your revised manuscript:

- A rebuttal letter that responds to each point raised by the academic editor and reviewer(s). You should upload this letter as a separate file labeled 'Response to Reviewers'.
- A marked-up copy of your manuscript that highlights changes made to the original version. You should upload this as a separate file labeled 'Revised Manuscript with Track Changes'.
- An unmarked version of your revised paper without tracked changes. You should upload this as a separate file labeled 'Manuscript'.

**Response:** We are submitting the rebuttal letter containing each point raised by the academic editor and reviewers.

We are also sending one copy of the ‘Revised Manuscript with Track Changes’ and one clean copy of the revised version labeled as ‘Manuscript’.

**Comment:** If you would like to make changes to your financial disclosure, please include your updated statement in your cover letter.

**Response:** Required modifications in the financial disclosure has included in the updated statement of the cover letter.

**Comment:** Guidelines for resubmitting your figure files are available below the reviewer comments at the end of this letter.

**Response:** We are resubmitting the figure files according to the guidelines of this journal.

**Reviewers’ comments:**

**Reviewer's Responses to Questions:**

**Comment:** 1. If the authors have adequately addressed your comments raised in a previous round of review and you feel that this manuscript is now acceptable for publication, you may indicate that here to bypass the “Comments to the Author” section, enter your conflict of interest statement in the “Confidential to Editor” section, and submit your "Accept" recommendation.

Reviewer #1: All comments have been addressed

2. Is the manuscript technically sound, and do the data support the conclusions?
The manuscript must describe a technically sound piece of scientific research with data that supports the conclusions. Experiments must have been conducted rigorously, with appropriate controls, replication, and sample sizes. The conclusions must be drawn appropriately based on the data presented.

Reviewer #1: Partly

3. Has the statistical analysis been performed appropriately and rigorously?

Reviewer #1: N/A

4. Have the authors made all data underlying the findings in their manuscript fully available?
The [PLOS Data policy](http://www.plosone.org/static/policies.action#sharing) requires authors to make all data underlying the findings described in their manuscript fully available without restriction, with rare exception (please refer to the Data Availability Statement in the manuscript PDF file). The data should be provided as part of the manuscript or its supporting information, or deposited to a public repository. For example, in addition to summary statistics, the data points behind means, medians and variance measures should be available. If there are restrictions on publicly sharing data—e.g. participant privacy or use of data from a third party—those must be specified.

Reviewer #1: Yes

5. Is the manuscript presented in an intelligible fashion and written in standard English?
PLOS ONE does not copyedit accepted manuscripts, so the language in submitted articles must be clear, correct, and unambiguous. Any typographical or grammatical errors should be corrected at revision, so please note any specific errors here.

Reviewer #1: Yes

**Response:** Thank you so much for your appreciation and positive responses.

**Review Comments to the Author:**

**Reviewer #1:**

Clearly authors have tried to address some of the issues with the paper and overall the paper is improved. I am afraid that I do not agree with the author’s statement that Y132F is always accompanied by C461T. Indeed, there are numerous cases in which Y132F (A395T) does occurs alone without C461T. As suggested before, this will create a significant challenge to the HRM technique, since a) Y132F is resulted from substitution of A to T, therefore it will not have that much influence on the melting temperature of the amplicons with only Y132F, and b) the fact that Y132F can occur alone preclude the usage of this technique as a comprehensive surrogate marker. Therefore, the collection of fluconazole-resistant C. tropicalis isolates used by authors will not dictate what can be found in other centers, <https://www.mdpi.com/2309-608X/6/3/138.> Moreover, in our laboratory we have used a lot of times to adjust the TM difference between susceptible and resistant isolates as far as possible (at least more than one degree of centigrade) to prevent misidentification and we have witnessed cases, where amplicons with a difference of one degree of centigrade were misidentified when tested in the other labs, which is inherent and may arise due to change in setting, reagent, the DNA extraction method, the purity of the DNA samples, etc. Finally, the TM difference authors observed is just a fraction of centigrade and it can cause misidentification in isolates carrying A395T+C461T and will not differentiate those only carrying A395T. The same concern is also applicable to restriction site mutation approach, since it only cut C461T and cannot differentiate resistant isolates carrying only Y132F from susceptible isolates. It is understandable that authors spent time, energy, and money to develop these assays, but the final outcome of the technique should be considered. If authors still advocate their statements, they are encouraged to mention these concerns as the pitfall of their techniques in discussion and call for improvements.
Our goal as a reviewer is to enhance the quality of the papers and do hope that authors comprehend that.

**Response:** Thank you so much for your meticulous comment and valuable suggestion. As per your suggestion, we have mention these concerns as the pitfall of our techniques in discussion of the revised manuscript.

**Comment:** PLOS authors have the option to publish the peer review history of their article. If published, this will include your full peer review and any attached files.
If you choose “no”, your identity will remain anonymous but your review may still be made public.

**Response:** We have no problem to publish the peer review history of our article.

***We would like to thank the Academic Editor and Reviewers for their important comments and valuable suggestions that have been improved the manuscript significantly.***
